# *Pharm to Table* Podcast Duo Bridges the Academia–Industry Divide

**DOI:** 10.1021/acscentsci.6c00691

**Published:** 2026-04-24

**Authors:** Mark Peplow

## Abstract

The Merck colleagues and cohosts advocate closer collaboration between academic and industry chemists.

## Graphical abstract

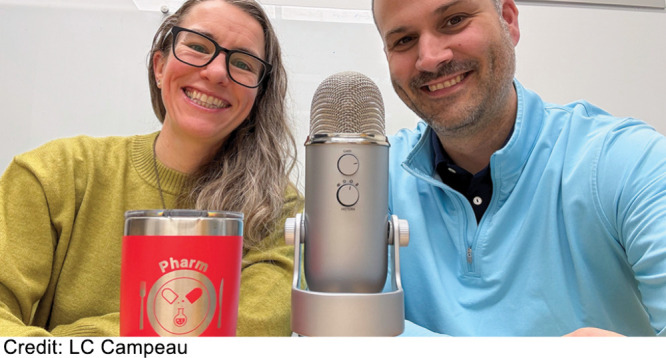

Sometimes it can feel like there’s a big gap between academic chemists and their counterparts in the pharmaceutical industry. Pharma chemists may seem slow to adopt new synthetic methods, for example, while academics might not be aware of constraints that limit a technique’s use in industry.

**Figure:**
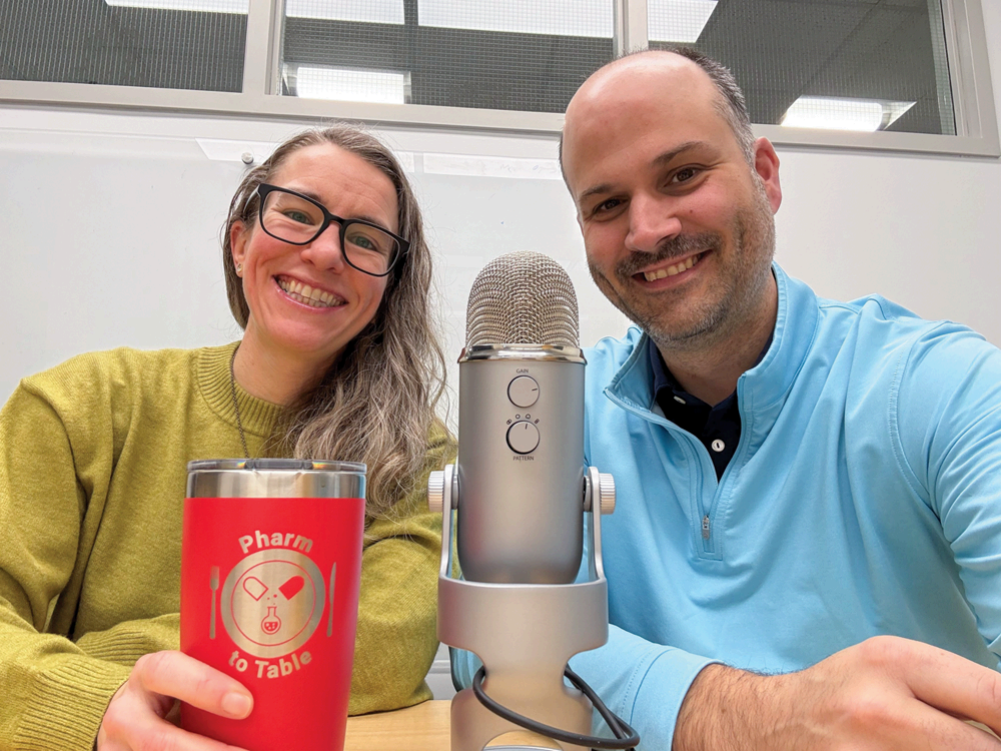
In addition to working day jobs as chemists at Merck & Co., Dani Schultz (left) and LC Campeau (right) host the *Pharm to Table* podcast to demystify the pharma industry. Credit: LC Campeau.

Back in 2020, Merck & Co. process chemists Dani Schultz and LC Campeau coauthored a provocative think piece in *Nature Chemistry*, arguing that this divide holds back innovation in drug development. “In order to increase the speed of translation of fundamental advances in chemistry into new medicines,” they wrote, “industrial scientists are going to have to roll up their sleeves and engage.” In the piece, they advocate a model in which academics collaborate much more closely with industry chemists, taking advantage of advanced industrial facilities to deliver practical solutions to key problems in drug development.

Today there’s much more openness to industry–academia collaboration, and such projects are clearly bearing fruit. Schultz and Campeau launched the *Pharm to Table* podcast in 2021 to showcase how these collaborations operate at Merck and to encourage further engagement from academic chemists by demystifying the chemistry that Merck scientists work on.

Mark Peplow spoke with Schultz and Campeau to discuss the changing landscape of academia–industry partnerships, the world of podcasting, and what they’ve learned along the way about communication, collaboration, and chemistry. This interview was edited for length and clarity.

## How has the relationship between academia and industry changed over the years?

**LC Campeau:** It used to be very transactional. Twenty years ago, pharma would just give money to academics to do great stuff, and they’d thank us in their article. That type of model doesn’t really exist anymore.

At Merck, we had evolved a model for collaborative research projects that was more akin to how academics collaborate with one another. Our goal for the *Nature Chemistry* article was to put that model out there and say it can have a tremendous impact on both the industrial side and the academic side.

It was a bit of a call to arms, and I think the message resonated because there was a broader movement headed in that direction already. Now basically every pharmaceutical company in the US does this.

**Dani Schultz:** I think that article helped people see that we’re approachable. A lot of early career scientists started coming up to me at meetings, saying, “Can you check out my poster?”

More recently, I’ve also seen academic presentations including a slide that says, “If industry is interested in partnering with me and my group, please reach out to me.” There’s more salesmanship, maybe because funding is so challenging these days.

## Can you give me an example of this kind of collaboration?

**DS:** Five or six years ago, we were thinking about ways to accelerate peptide drug discovery using late-stage functionalization [modifying a molecule close to the end of its synthesis]. That’s pretty well established in the small-molecule field, but not so much in peptides.

Mary Watson at the University of Delaware had developed a really versatile pyridinium cross-electrophile coupling, so we asked her if she’d ever thought about trying it with peptides.

What came out of that project was a pretty cool reaction. The pyridinium amino acid we used was very robust, and it could be loaded on to a peptide synthesizer, which allowed our medicinal chemists to rapidly adopt it.

**LCC:** What I love about that example is that it’s exactly what we wrote about in *Nature Chemistry*. One of the critical elements [of a successful collaboration] is problem selection—where’s the right place to use this cool innovation—but with the equally important step of asking how it will be implemented by pharma. If it’s very finicky and difficult to execute, then people won’t use it. But this one checked all the boxes, and we had massive uptake.

**Figure:**
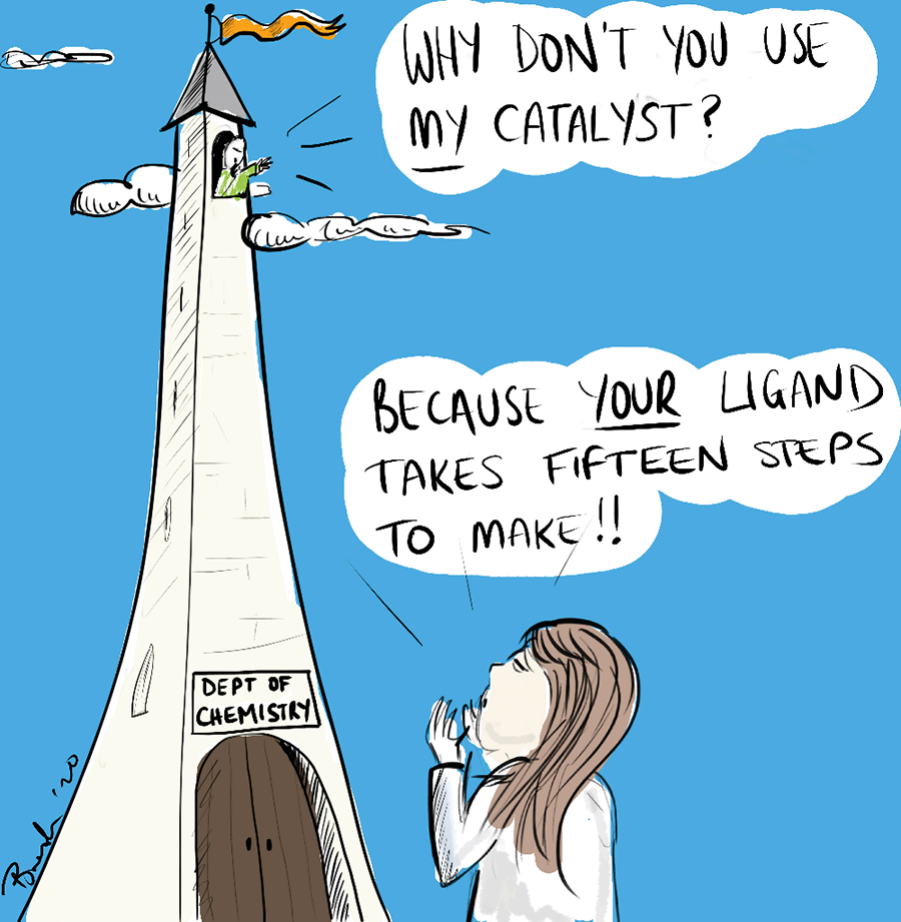
A cartoon panel from Campeau and Schultz’s *Nature Chemistry* commentary piece illustrates the gap that sometimes needs to be bridged between academia and industry. Credit: Courtesy of *Nature Chemistry* and Brendan Burkett (@ChemScrapes).

## What about other pharma companies? Have they seen success with this collaboration model?

**LCC:** There’s a long-standing collaboration between the Pfizer chemistry group and Dan Weix at the University of Wisconsin–Madison, which has led into areas that probably neither group would have done otherwise. In 2024, for example, they leveraged hardcore med chem computational methods to develop ligands for nickel catalysts and screened them for cross-electrophile coupling reactions.

I don’t think Dan could have done that on his own, because it’s just too big a project. And I don’t think Pfizer would have bothered if they didn’t already have that partnership.

## What other areas are ripe for academia–industry collaborations?

**DS:** Many peptide drugs are required on the metric ton scale, but a lot of the manufacturing processes they rely on are very dated. They’re inefficient, and they waste a lot of solvent.

For example, solid-phase peptide synthesis got the Nobel Prize in 1984, the year I was born, and 42 years later we’re still using the exact same resins that were employed back then. Those resins really limit our ability to make this a greener process, because they’re only suitable if you use solvents like DMF [dimethylformamide].

**LCC:** We’re getting to the point where a significant fraction of the world’s DMF is going into washing resins for peptides, which is just not sustainable.

**DS:** So if a polymer research group invented new resins that are tolerant of greener, safer, less toxic solvents, it would do a remarkable good for peptide manufacturing.

## Why did you start your podcast?

**LCC:** Dani and I have spent a lot of time in our careers traveling to universities, meeting with students, and sharing Merck science. It’s an important way to seed innovation, but it’s also a recruiting tool—you’re building relationships and giving people a sense of what it’s like to work at Merck.

Then COVID-19 happened, and shutdowns meant we couldn’t travel anymore. We realized that rather than visiting one school at a time, a podcast would allow us to reach anyone, anywhere, at any time, and continue our commitment to collaboration with the academic community. I don’t think I could be happier with how it has turned out.

**DS:** We also wanted to prominently feature our research collaborations, and have academic voices be part of it, so that other people could hopefully be inspired or learn something. Students and professors are always curious about what we do, so we try to get behind the scenes and discuss what’s not in the paper.

Many of our listeners are graduate students, so last year we did a two-part episode on how to get a career in industry. We invited on hiring managers from our discovery and process chemistry divisions and went through the questions that students always ask: What should be on your CV? What should your job talk focus on? What kind of questions are you going to be asked? Those episodes are our top-rated episodes.

## What have you learned about communication from your podcasting experience?

**DS:** Keep it short and sweet. People typically listen to these podcasts on a commute, so 30 min on average. We also try to make it lighthearted, which helps keep people engaged.

**LCC:** It has helped me think more about the audience when crafting a message. If you do a PhD, you’re used to giving talks, but a lot of us weren’t trained to think about who’s receiving your message.

## Would you ever consider hosting a popular-science podcast, to reach an audience of nonchemists?

**DS:** Because you asked, I’m just going to put this into the ether. So, Bowen Yang is a comedian who was on *Saturday Night Live*, and he has a bachelor’s in chemistry from NYU.

But he’s not on *SNL* anymore, and I’m just thinking, maybe he has some time to do some chemistry podcasting? Let’s talk about bringing chemistry to everyone and making it fun and approachable. So, Bowen, if you read this, you know where to find us!

*Mark Peplow is a freelance contributor to* Chemical & Engineering News *, an independent news publication of the American Chemical Society.*

